# Unveiling Barriers to the Modern Contraceptive Uptake in the Urban Slums of Karachi: Perceptions, Attitude, and Accessibility

**DOI:** 10.7759/cureus.56164

**Published:** 2024-03-14

**Authors:** Ambareen Main Thompson, Justin Main Thompson, Hina Sharif, Tooba Seemi, Sana Sharif

**Affiliations:** 1 Department of Public Health, King's College London, London, GBR; 2 Department of Public Health, University of Salford, London, GBR; 3 Department of Research and Publication, SINA Health, Education & Welfare Trust, Karachi, PAK; 4 Department of Community Health Sciences, Aga Khan University Hospital, Karachi, PAK; 5 School of Public Health, University of Saskatchewan, Toronto, CAN

**Keywords:** oral contraceptive pill (ocp), iud, unplanned pregnancy, contraceptive, condom, family planning, contraception

## Abstract

Background

Modern contraception plays a vital role in family planning and preventing unintended pregnancies. However, its uptake remains limited in many developing countries, including Pakistan. This study aimed to evaluate the barriers to modern contraception and identify strategies to enhance its adoption in the urban slums of Karachi.

Methods

A multi-site, cross-sectional study was conducted in 38 slum areas of Karachi, Pakistan. Women aged 15-49 years were interviewed using a comprehensive questionnaire. The questionnaire covered socio-ethnic and economic demographics, knowledge and perceptions of modern contraception, accessibility, affordability, attitudes, and usage. Data analysis was performed using the Statistical Package for Social Sciences (SPSS) version 24 (IBM SPSS Statistics, Armonk, NY).

Results

The majority of the respondents identified as Pathan ethnicity (49%), and the age range was predominantly from 23 to 34 years (45.5%). A high proportion of participants demonstrated satisfactory knowledge of contraceptives (87.6%). However, a significant portion perceived contraception or family planning to be in conflict with religious beliefs (84%). Many women expressed a desire for more children (56%) and had concerns about contraceptive side effects (78%). A notable proportion of women reported that their spouses forbade the use of contraceptives (12%). Among the surveyed population, the most widely used contraceptives were injections among women (15.5%) and condoms among their male partners (12%).

Conclusion

Despite sufficient knowledge and accessibility, considerable barriers exist in the uptake of modern contraception in the urban slums of Karachi, Pakistan. These barriers include religious conflicts, cultural norms, concerns about side effects, spousal disapproval, and desires for larger families.

## Introduction

Nationally, Pakistan faces significant challenges in ensuring widespread access to modern contraceptives. The contraceptive prevalence rate in the country remains low, with only a fraction of women using modern methods of contraception [1]. The unmet need for family planning is high, indicating the demand for contraceptives that remains unfulfilled [2]. The government of Pakistan has recognized the importance of family planning and has implemented various initiatives to improve access to contraceptives. However, these efforts often face implementation challenges and may not effectively reach women in urban slums [3].

The uptake of modern contraceptives plays a crucial role in promoting family planning, improving maternal and child health, and empowering women to make informed decisions about their reproductive health [4]. However, in the urban slums of Karachi, Pakistan, there exist numerous barriers that hinder the adoption of modern contraceptives. Karachi, the largest city in Pakistan, is characterized by rapid urbanization and a high population density. Within this urban landscape, slums have emerged as marginalized communities with limited access to basic services, including healthcare [5]. These urban slums are densely populated, lack proper sanitation facilities, have limited infrastructure, and experience high levels of poverty [6]. These conditions exacerbate the reproductive health challenges faced by women residing in these areas.

The prevalence of unintended pregnancies and maternal mortality rates in the urban slums of Karachi is disproportionately high compared to the general population [7]. Limited access to contraception contributes to these adverse outcomes. Women in these areas face numerous barriers to accessing modern contraceptives, including social and cultural norms, the lack of awareness, financial constraints, and the limited availability of contraceptive services [8]. Understanding these barriers is essential for designing effective interventions to address the specific needs of women in urban slums.

The available data indicating the need for the study to identify the issues in contraceptive uptake in the urban slums of Karachi include low contraceptive prevalence rates, high unmet need for family planning, and a reliance on traditional methods of contraception, which are often less effective [9]. The lack of awareness and misconceptions surrounding modern contraceptives further hinder their adoption [10]. Additionally, the limited availability of contraceptive services, such as clinics and trained healthcare providers, restricts access to contraceptives for women in these communities [11].

Internationally, organizations such as the World Health Organization (WHO) and the United Nations Population Fund (UNFPA) emphasize the importance of family planning and access to contraceptives as essential components of reproductive health and women's empowerment [12]. The Sustainable Development Goals (SDGs) set by the United Nations include targets related to universal access to sexual and reproductive healthcare, including family planning services [13]. However, the urban slums of Karachi present a unique context with specific challenges that require localized research and interventions.

The rationale for conducting this study stems from the urgent need to address the barriers to modern contraceptive uptake in the urban slums of Karachi. By gaining insights into the perceptions, attitudes, and accessibility issues related to contraceptives, we can develop evidence-based interventions tailored to the specific needs of women in these communities. Hence, this study aims to unveil these barriers and explore strategies to enhance the uptake of modern contraceptives in this specific context. By investigating the perceptions, attitudes, and accessibility of contraceptives, we can identify effective interventions to address the challenges faced by women in urban slums. This study will contribute to the existing body of knowledge by providing context-specific findings that can inform policy-making and programmatic efforts aimed at improving reproductive health outcomes in urban slums.

Objectives of the study

The objectives of the study are as follows: to determine the magnitude and factors contributing to the use of contraception among women of reproductive age residing in the urban slums of Karachi, Pakistan; to explore the multifaceted barriers and challenges encountered by women in the urban slums of Karachi, Pakistan, in accessing and effectively utilizing contraceptive methods; and to examine the influence of demographic characteristics (such as age, marital status, and parity) and socio-economic factors (including education, income, and healthcare access) on the use of contraception among women in the urban slums of Karachi, Pakistan.

## Materials and methods

Study type

This is a descriptive, cross-sectional study.

Study location

The study was conducted in 38 low-income slum communities in Karachi, Pakistan, which is the largest city in the country and is known for its rapid urbanization. Karachi is a bustling metropolis with a diverse population and is home to a significant proportion of the country's population.

The study specifically targeted low-income slum communities within Karachi. The selected slum communities represented a range of different areas within Karachi, including Shireen Jinnah, Baldia Town, Machar Colony, Yousuf Sahab Goth, Yousuf Arfani Goth, Mewashah, Hassan Noman Town, Gujro Gadap, Jamali, Mehran Town, Bilal Colony, Korangi Ittehad, Jumma Goth, Landhi, Sherpao Colony, and Moach Goth. This diverse selection aimed to capture the varied socio-economic and cultural contexts of slum communities in Karachi.

It is worth noting that the study was conducted in collaboration with a primary healthcare facility that provided highly subsidized modern contraception services to the target population. This ensured that the participants had access to quality contraceptive options and received medical guidance from qualified healthcare practitioners.

Sample size

The prevalence of unmet needs for contraception is not specified in underserved, marginalized populations. In such cases, a conservative estimate of 0.5 (50%) can be used to determine the maximum required sample size.

Using a 95% confidence level and a margin of error of 5%, the calculated sample size is approximately 384. With a nonresponse rate of 10%, the sample size inflated to 423.

Study population

The study population comprised 444 women in the reproductive age group who participated in the study. These women were residing in urban slum areas across 20 different locations in Karachi, Pakistan. Eligibility criteria are presented in Table 1.

**Table 1:** Eligibility criteria of the participants in the study

Inclusion criteria	Exclusion criteria
Women of reproductive age, typically defined as 15-49 years old	Women who are not within the reproductive age group (below 15 years or above 49 years)
Residing in the urban slum areas of Karachi, Pakistan	Residing outside the designated urban slum areas of Karachi
Willingness to participate in the study and provide informed consent	Refusal or inability to provide informed consent for participation
Ability to understand and respond to the interview questions in the selected language(s)	Inability to communicate effectively in the selected language(s) to answer the interview questions
Visiting or seeking healthcare services at the primary healthcare facility providing highly subsidized modern contraception services in the study areas	Not visiting or seeking healthcare services at the primary healthcare facility providing highly subsidized modern contraception services in the study areas
Women of reproductive age, defined as 15-49 years old	

Sampling procedure

The sampling procedure for this study involved a multistage cluster sampling method to select the study participants from urban slum areas in Karachi, Pakistan. The following steps were followed to ensure a scientifically sound and methodologically correct sampling approach.

Selection of Slum Areas

A comprehensive list of urban slum areas in Karachi was obtained from local authorities and relevant community organizations. The slum areas were categorized based on their geographic location and population size. The study specifically targeted low-income slum communities within Karachi. They are often marginalized and face numerous socio-economic challenges. The selected slum communities represented a range of different areas within Karachi, including Shireen Jinnah, Baldia Town, Machar Colony, Yousuf Sahab Goth, Yousuf Arfani Goth, Mewashah, Hassan Noman Town, Gujro Gadap, Jamali, Mehran Town, Bilal Colony, Korangi Ittehad, Jumma Goth, Landhi, Sherpao Colony, and Moach Goth. This diverse selection aimed to capture the varied socio-economic and cultural contexts of slum communities in Karachi.

Stratum Formation

The slum areas were divided into strata based on their respective categories. This division was done to account for the heterogeneity among slum areas in terms of socio-economic status, infrastructure, and accessibility to healthcare services.

Random Selection of Slum Areas

From each stratum, a proportionate number of slum areas were randomly selected to ensure representation from different categories. The number of selected slum areas was determined based on the overall sample size and the distribution of the population across the slum areas.

Identification of Eligible Participants

Within the selected households, women in the reproductive age group (15-49 years) were identified as potential study participants. Their eligibility was assessed based on the inclusion criteria, including residency in the selected slum areas and willingness to participate in the study.

Informed Consent and Data Collection

Eligible women were approached, and the study objectives, procedures, and potential risks and benefits were explained to them. Informed consent was obtained from those willing to participate. Structured interviews using a questionnaire were conducted to collect the required data, ensuring privacy and confidentiality.

Achieving the Desired Sample Size

We tried to collect data on the sample size of 423 women already determined based on the desired confidence level, margin of error, and estimated prevalence or effect size. During the process, data on 444 eligible women were collected.

Data collection procedure

The data collection procedure for this study involved conducting structured interviews with the selected participants using a comprehensive questionnaire. The following steps were followed to ensure a systematic and standardized approach to data collection.

Questionnaire Development

A semi-structured questionnaire was developed to collect data on various aspects related to contraception utilization and unmet needs. The questionnaire was designed based on a review of relevant literature, consultation with experts, and the consideration of the study objectives. It encompassed demographic characteristics, economic status, ethnicity, educational background, number of children, knowledge about modern contraception, availability and affordability of contraceptive methods, barriers to contraception usage, and reasons influencing the decision to use or not to use contraceptives.

Training of Interviewers

The interviewers involved in data collection underwent a comprehensive training program to ensure a standardized approach. They were trained on the study objectives, ethical considerations, interview techniques, and the use of the questionnaire. The training also focused on maintaining privacy and confidentiality, establishing rapport with participants, and handling sensitive topics.

Pilot Testing

Before the actual data collection, a pilot study was conducted to test the questionnaire and assess its clarity, comprehensibility, and suitability for the target population. The pilot study involved a small sample (n=20) of participants similar to the study population. Then, Cronbach's alpha was estimated for validity, which is 0.8.

Informed Consent

Prior to the interview, verbal informed consent was obtained from each participant. The purpose and objectives of the study, the voluntary nature of participation, and the confidentiality of the data were explained to the participants. They were assured that their participation was voluntary, and they could withdraw at any time without any consequences.

Data Collection

The interviews were conducted in a private and comfortable setting to ensure confidentiality. The interviewers followed the standardized questionnaire, asking the questions and recording the responses accurately. They maintained a nonjudgmental and supportive approach throughout the interviews, allowing the participants to express their opinions and experiences freely.

Quality Control

To ensure data quality and consistency, regular supervision and monitoring of the data collection process were carried out. The supervisors provided guidance and support to the interviewers, addressed any queries or concerns, and conducted periodic checks of the collected data for completeness and accuracy.

Data Management

The collected data were securely stored and managed in a confidential manner. Data entry was performed using appropriate software, ensuring accuracy and reliability. Data cleaning and validation procedures were implemented to identify and resolve any discrepancies or errors.

Ethical Considerations

The study followed ethical guidelines to protect the rights and well-being of the participants. The confidentiality and privacy of the participants' information were strictly maintained. The proposal was presented to SINA's Ethical Review Board (SINA-ERB) before the initiation of the study, which granted its approval via ERB number ERB0000014/03-23.

Patient and Public Involvement

No patients or members of the general public were involved in the planning, execution, reporting, or distribution of our study.

Statistical analysis

The collected data were carefully entered into Microsoft Excel (Microsoft Corp., Redmond, WA) and underwent a rigorous process of quality checks to ensure accuracy and consistency. To assess the relationships between contraceptive use and sociodemographic factors, a bivariate analysis was conducted. Variables were screened based on their p-values, with a significance threshold of <0.20, to determine which variables should be included in the multivariate analysis model.

In the multivariate analysis, a logistic regression model was employed to examine the significant associations between the outcome variable (contraceptive use) and the predictor variables. Only the predictor variables that demonstrated statistical significance in the bivariate analysis were included in the multivariate analysis. This approach allowed for the evaluation of the independent contributions of the selected predictor variables while controlling for potential confounding factors.

The use of multivariate logistic regression enabled us to identify the significant factors associated with contraceptive use while accounting for the interplay of multiple variables. This statistical technique allowed for a more comprehensive understanding of the factors influencing contraceptive use among women in the urban slum areas of Karachi.

It is important to note that statistical analyses were conducted using the Statistical Package for Social Sciences (SPSS) version 24 (IBM SPSS Statistics, Armonk, NY), a widely used software package for data analysis. The significance level was set at p<0.05 to determine statistical significance. The results of the analysis were interpreted, considering the odds ratios, confidence intervals (CI), and corresponding p-values, to assess the strength and direction of the associations between the predictor variables and contraceptive use.

## Results

The results of the bivariate analysis examining the association between contraceptive use and sociodemographic factors among women in the urban slums of Karachi, Pakistan, are presented in Table 2. A total of 444 women participated in the study.

**Table 2:** Contraceptive method: users and nonusers' descriptive analysis (N=444) A bivariate analysis using p-values with a significance threshold of <0.20

Variables	Total	Are you or your husband currently using any contraception method?		p-value
		N (%)	N (%)	
		Yes	No	
Age	≤24	11 (2.4)	39 (8.7)	0.569
	25-34	60 (13.5)	142 (31.9)	
	≥35	48 (10.8)	144 (32.4)	
Occupation	Housewife	113 (25.4)	298 (67.1)	0.2
	Working women	6 (1.3)	27 (6)	
	Total	119 (26.7)	325 (73.2)	
Monthly income	10,000-30,000	89 (20)	186 (41.8)	0.001
	30,000-50,000	2 (0.4)	7 (1.57)	
	Less than 10,000	27 (6)	124 (27.9)	
	No income	1 (0.2)	8 (1.8)	
	Total	119 (26.8)	325 (73.2)	
Religion	Muslim	113 (25.4)	317 (71.3)	0.769
	Non-Muslim	6 (1.35)	8 (1.8)	
	Total	119 (26.8)	325 (73.1)	
Educational level	Bachelors	2 (0.4)	5 (1.1)	0.1
	Intermediate	4 (0.9)	6 (60)	
	No education	90 (20.2)	275 (61.9)	
	Primary	10 (2.25)	22 (4.9)	
	Secondary	13 (2.9)	17 (3.8)	
	Total	119 (26.8)	325 (73.2)	
Ethnicity	Afghani	5 (1.1)	23 (5.1)	0.011
	Baloch	3 (0.6)	16 (3.6)	
	Bangali	2 (0.4)	12 (2.7)	
	Christian	2 (0.4)	1 (0.2)	
	Pathan	58 (13)	161 (36.2)	
	Punjabi	16 (3.6)	57 (12.8)	
	Sindhi	19 (4.2)	39 (8.7)	
	Urdu speaking	14 (3.1)	16 (3.6)	
	Total	119 (26.4)	325 (72.9)	
Do you think that contraception is against any religious or cultural beliefs?	Yes	78 (17.5)	295 (66.4)	0.00
	No	41 (9.2)	30 (6.7)	
	Total	119 (26.7)	325 (73.1)	
Do you think that using contraception is safe for women?	Yes	56 (12.6)	40 (9)	0.00
	No	63 (14.1)	285 (64.1)	
	Total	119 (26.8)	325 (73.1)	
Do you have any concern about the cost of contraception?	Never used	21 (4.7)	262 (59)	0.00
	No	98 (22)	63 (14.1)	
	Total	119 (26.7)	325 (73.1)	
Are you currently pregnant or trying to be pregnant?	Currently pregnant	3 (0.6)	75 (16.8)	0.001
	No	113 (25.4)	177 (39.8)	
	Trying to be pregnant	3 (0.6)	73 (16.4)	
	Total	119 (26.6)	325 (73)	
Do you want to have more children in the future?	Yes	46 (10.3)	203 (45.7)	0.00
	No	73 (16.4)	122 (27.4)	
	Total	119 (26.7)	325 (73.1)	

Age was not found to be significantly associated with contraceptive use (p=0.569). The distribution of contraceptive use was similar across different age groups, with 2.4% of women aged ≤24, 13.5% of women aged 25-34, and 10.8% of women aged ≥35 reporting current use of contraception. Occupation also did not show a significant association with contraceptive use (p=0.2). Among the participants, 25.4% of housewives and 1.3% of working women reported using contraception. Monthly income demonstrated a significant association with contraceptive use (p=0.001). A higher proportion of women with a monthly income of 10,000-30,000 (20%) reported using contraception compared to those with a monthly income of 30,000-50,000 (0.4%), less than 10,000 (6%), or no income (0.2%). Religion, educational level, and ethnicity did not show significant associations with contraceptive use (p>0.05). Among the participants, 25.4% of Muslim women and 1.35% of non-Muslim women reported using contraception. Furthermore, contraceptive use varied across different educational levels and ethnicities, but these differences were not statistically significant. Perceptions regarding religious or cultural beliefs and the safety of contraception showed significant associations with contraceptive use (p<0.001). Women who believed that contraception is against religious or cultural beliefs (17.5%) were less likely to use contraception compared to those who did not hold this belief (9.2%). Similarly, a lower proportion of women who perceived contraception as safe (12.6%) reported using contraception compared to those who did not perceive it as safe (14.1%). Cost concerns regarding contraception were significantly associated with contraceptive use (p<0.001). Women who had never used contraception (4.7%) were less likely to report cost concerns compared to those who had used contraception (22%). Pregnancy status and the desire for more children were both significantly associated with contraceptive use (p<0.001 and p<0.05, respectively). A higher proportion of currently pregnant women (0.6%) reported using contraception compared to those who were not pregnant (25.4%) or were trying to be pregnant (0.6%). Additionally, women who expressed a desire for more children (10.3%) were less likely to use contraception compared to those who did not desire more children (16.4%).

The results of the multivariate logistic regression analysis are presented in Table 3. According to the results, occupation was significantly associated with contraceptive use (p=0.005). Women who were engaged in occupations other than housewives had higher odds of using contraception (adjusted odds ratio {AOR}=1.195 and 95% {CI}=1.055-1.353) compared to housewives. The belief that contraception is against religion and culture was also significantly associated with contraceptive use (p=0.038). Women who held this belief had higher odds of using contraception (AOR=1.134 and 95% CI=1.036-1.241) compared to those who did not hold this belief. Cost factor was found to be significantly associated with contraceptive use (p<0.001). Women who reported cost concerns regarding contraception had lower odds of using contraception (AOR=0.639 and 95% CI=0.592-0.689) compared to those who did not have cost concerns. The perception of contraceptive safety for women's health showed a significant association with contraceptive use (p<0.001). Women who perceived contraception as not safe had lower odds of using contraception (AOR=0.786 and 95% CI=0.716-0.864) compared to those who perceived it as safe. Pregnancy status and the desire for pregnancy were also significantly associated with contraceptive use (p=0.047 and p=0.006, respectively). Women who were currently pregnant had lower odds of using contraception (AOR=0.962 and 95% CI=0.865-1.070) compared to those who were not pregnant. On the other hand, women who were trying to become pregnant had higher odds of using contraception (AOR=1.134 and 95% CI=1.036-1.241) compared to those who were not trying to become pregnant.

**Table 3:** The multivariate logistic regression analysis among the significant predictors A logistic regression model for analyzing the significant correlations between contraceptive usage and the predictor variables in the multivariate analysis. Only bivariate significant predictor variables were included in the multivariate analysis (p=0.005) AOR, adjusted odds ratio; CI, confidence interval

Variables	Yes/no	AOR	95% CI	p-value
Employed	No	1.195	1.055-1.353	0.005
	Yes	Reference	-	-
Is contraception against religion and culture?	Yes	1.134	1.036-1.241	0.038
	No	Reference	-	-
Cost factor	Yes	0.639	0.592-0.689	0
	No	Reference	-	-
Is contraception safe for women's health?	No	0.786	0.716-0.864	0
	Yes	Reference	-	-
Currently pregnant or trying to become pregnant	Yes	0.962	0.865-1.070	0.047
	No	1.134	1.036-1.241	0.006
	Trying to be pregnant	Reference	-	-

**Figure 1:**
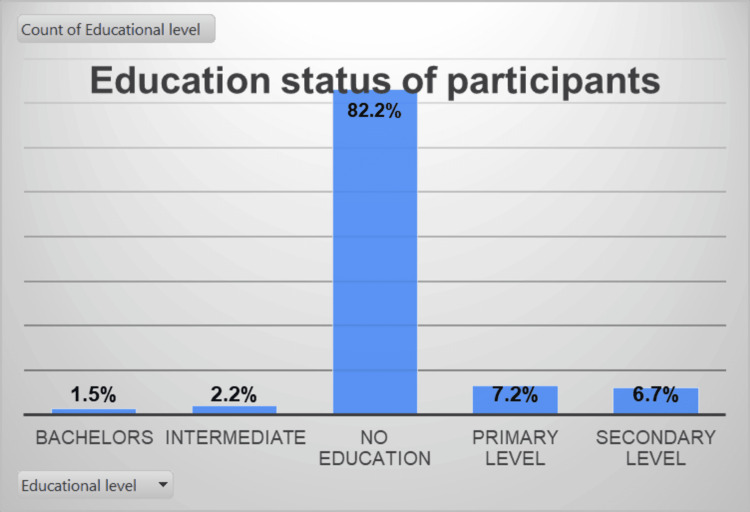
The educational status of the target population, the urban slum population of Karachi, Pakistan

The data presented in Figure 1 reveal a notable trend where the educated respondents displayed greater receptiveness toward contraception. This finding highlights the positive impact of education on contraceptive uptake and highlights the importance of promoting educational opportunities for women in urban slums.

However, it is essential to recognize the significance of women with no formal education, many of whom were Quran literate. They demonstrated the utilization of contraception based on their personal needs. This finding challenges the notion that cultural and religious barriers may impede contraceptive use among this population. It emphasizes the importance of understanding individual circumstances and preferences when addressing family planning needs, regardless of educational background.

Table 4 presents the frequencies of uptake and the knowledge of different types of contraception among the study participants. The percentages indicate the proportion of respondents who reported using or having knowledge about each type of contraception. Among the participants, condoms were the most commonly used method of contraception, with an uptake of 11.9%. Knowledge about condoms was relatively high, with 66.4% of the respondents indicating familiarity with this method. Contraceptive pills were reported by 1.6% of the participants as their chosen method of contraception, while 81.1% had knowledge of this option. Intrauterine devices (IUDs) were used by 12.0% of the respondents, with 23.3% having knowledge about this contraceptive method. Contraceptive patches were utilized by 28.5% of the participants, but only 1.7% reported having knowledge about this method. Periodic abstinence, a method involving abstaining from sexual intercourse during specific periods of the menstrual cycle, was reported by 10.8% of the participants. However, 98.1% of the respondents demonstrated knowledge of this contraceptive approach. Injectables were chosen by 7.2% of the participants, while 25.1% had knowledge of this method. Implants were utilized by 2.8% of the respondents, and only 0.8% had knowledge about this contraceptive option. A small proportion of participants (3.6%) indicated that they had never heard of any of the listed contraceptive methods. Additionally, 12.8% of the respondents reported not having knowledge about any of the contraception types mentioned in Table 3. These findings provide valuable insights into the uptake and knowledge of various contraceptive methods among women in urban slums. Understanding the patterns and gaps in contraceptive utilization and awareness is crucial for tailoring effective family planning interventions and promoting informed decision-making regarding contraception.

**Table 4:** Perception and uptake of available modern contraceptives *Intrauterine device (IUD) **Perceptions: beliefs, convenience, and side effects associated with contraception

Variables	Uptake of the type of contraception	Knowledge of the type of contraception
	Frequencies (%)	Frequencies (%)
Condoms	53 (11.9)	295 (66.4)
Contraceptive pills	7 (1.6)	360 (81.1)
*IUD	30 (12.0)	108 (23.3)
Contraceptive patches	71 (28.5)	8 (1.7)
**Periodic abstinence	27 (10.8)	430 (98.1)
Injectables	18 (7.2)	113 (25.1)
Implants	7 (2.8)	3 (0.8)
Never heard	9 (3.6)	57 (12.8)

**Figure 2:**
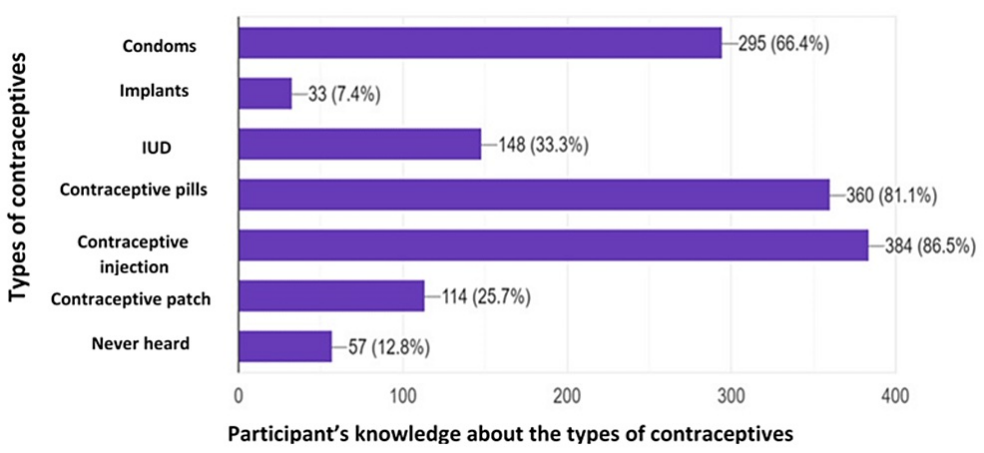
Types of contraception people of urban slums have heard of The total number of participants is 444 IUD: intrauterine device

Figure 2 presents a visual representation of the awareness levels regarding different contraceptive methods among individuals residing in urban slums. The figure offers a graphical depiction of the percentage of respondents who have heard of each contraceptive method.

## Discussion

The utilization of contraception, whether traditional or modern, is fundamental to effective family planning. Access to modern contraceptives, along with awareness and counseling on family planning, has played a significant role in the development and economic progress of both developed and developing nations. Many developing countries have implemented family planning programs, making modern contraceptives easily accessible and affordable to their populations. These programs also emphasize education and counseling on family planning to empower individuals in making informed decisions.

However, despite these efforts, several developing countries, including Pakistan, continue to face challenges in promoting the uptake of family planning and modern contraception. A study conducted by the Sustainable Development Policy Institute (SDPI) in 2004 [14] identified various factors contributing to this issue, including religious beliefs, resistance from husbands, cultural barriers, the lack of education, and the limited empowerment of women. While these factors have been identified, there is a need for a comprehensive quantitative study to explore them in greater detail. The study conducted by the Guttmacher Institute highlighted religion as one of the most influential factors contributing to the low uptake of contraception in Pakistan [15]. This finding aligns with a government survey conducted in 1990-1991, which also identified religious beliefs as a barrier to family planning.

In our study, a significant number of respondents expressed the belief that contraception was against their religious beliefs, accounting for 84% of the participants. Additionally, a considerable proportion of respondents reported never using any form of contraception. Among the respondents who were open to contraception, condoms were the preferred choice for 11.9% of them, followed by injectables favored by 6.3%. The utilization of contraception pills was relatively low. Conversations with respondents highlighted the influence of family pressure and the opinions of extended family members, such as mothers-in-law, on the decision-making process regarding contraception usage. Injectables were often chosen for their discreetness and longer duration of effectiveness. These findings indicate that barriers such as cultural and family influences continue to hinder the uptake of contraception [16,17].

During the study, two indicators of unmet need for family services in the slums emerged, one of which was the education level of women. A significant percentage of women reported having no formal education, limiting their awareness of the benefits of family planning. In their perspective, having a higher number of children was seen as a source of economic and social security in later life. This pattern is not unique to Pakistan and is observed in many underdeveloped or developing countries.

Research conducted by Kim supports this notion [18], showing that women with primary education have significantly fewer children compared to those with no education, and women with secondary education have even fewer children. This reduction in birth rates can be attributed to factors such as economic empowerment and a focus on quality investments in children. Bangladesh is an excellent example of the positive impact of female education on birth rates, with studies showing a decline in fertility rates alongside the rise of female education. Education brings about improved socio-economic factors, a better understanding of the benefits of family planning, and a desire to invest in children's future [19]. Other studies have also highlighted the crucial role of education, particularly female education, in reducing birth rates and improving reproductive health outcomes [20].

Iran, another Muslim country in the developing world, has also made significant progress in reducing its fertility rate. A report by the United States Institute of Peace (USIP) indicates that female literacy has substantially increased, and the average number of births per woman has decreased [21]. This progress can be attributed to the significant improvements in female education and the increased use of contraception. Ethiopia, a country in Africa, has observed similar results, with a study demonstrating a reduction in high-risk fertility behavior (HRFB) among women with higher levels of education [22].

Religious beliefs significantly influence the unmet need for contraceptives in the slums of Pakistan, particularly among the Muslim population. Many women in the study believed that contraception was against the principles of Islam and considered accepting as many children as God gave them as their moral responsibility. Similar findings have been observed in other studies conducted in Tanzania, where both Muslim and Christian respondents rejected family planning due to religious beliefs. These beliefs contribute to high fertility rates and socio-economic challenges, particularly for women [23].

While there are arguments within Islam supporting the permissibility of birth spacing, resistance to contraception continues to persist [24], particularly among women belonging to the Pashtun ethnicity. Despite the presence of strong arguments, cultural and religious factors continue to play a significant role in shaping attitudes toward contraception [25,26].

In conclusion, the challenges in promoting the uptake of family planning and modern contraception in the urban slums of Pakistan are influenced by various factors, including religious beliefs, cultural barriers, the lack of education, and the limited empowerment of women. Strategies to address these challenges should include engaging religious and community leaders, promoting education and awareness, and providing access to modern contraception and counseling services. Lessons can be learned from the experiences of other countries, where efforts to improve female education and empowerment have resulted in reduced birth rates and improved reproductive health outcomes. By prioritizing education, addressing cultural and religious beliefs, and providing comprehensive family planning services, we can work toward overcoming these challenges and promoting informed decision-making regarding contraception in urban slum populations.

Strengths

The research may highlight the promising strategies for improving contraception use in underserved populations through this study as this study highlights the knowledge status and barriers of the unmet need for contraceptives.

Limitations

The study encountered several limitations that should be taken into consideration. Primarily, the lack of formal education among many women in the study posed challenges to their comprehension ability, which affected their ability to provide specific details about the contraception methods they used. This limitation highlights the need for improved educational opportunities to empower women and enhance their understanding of reproductive health.

Also, perceptions and language barriers were evident among the participants, further hindering their ability to provide detailed information. This limitation suggests the importance of culturally sensitive approaches and the use of appropriate language in research and healthcare interventions to ensure effective communication and understanding.

It is also worth noting that women's limited understanding of certain procedures, such as the placement of an intrauterine device (IUD), tubal ligation, or abortion, was reflected in their use of the term "operation" to refer to these methods. This indicates the need for educational efforts to improve women's knowledge about different contraceptive options and reproductive health services.

While some indications of gender preference and the lack of bodily autonomy were observed in the study, the scope of the research did not allow for an in-depth exploration of these complex issues. Further research is needed to delve into these topics and understand the underlying factors influencing women's decision-making processes regarding contraception.

## Conclusions

The study highlights the persistent challenges in achieving satisfactory levels of modern contraception uptake in Karachi's slums. To address these challenges, a multifaceted approach is required, involving counseling services, social media platforms, the engagement of religious influencers, and prioritizing education for women. By taking prompt and comprehensive action, Pakistan can make significant progress in promoting family planning, reducing fertility rates, and improving reproductive health outcomes in urban slum communities.
